# Level of education, labor-market marginalization, and alcohol-related mortality: a cohort study of Swedish men

**DOI:** 10.1093/eurpub/ckaf163

**Published:** 2025-09-25

**Authors:** Emelie Thern, Tomas Hemmingsson, Emma Carlsson, Katarina Kjellberg, Melody Almroth

**Affiliations:** Institute of Environmental Medicine, Karolinska Institutet, Stockholm, Sweden; Department of Public Health Sciences, Stockholm University, Stockholm, Sweden; Institute of Environmental Medicine, Karolinska Institutet, Stockholm, Sweden; Department of Public Health Sciences, Stockholm University, Stockholm, Sweden; Department of Public Health Sciences, Stockholm University, Stockholm, Sweden; Department of Global Public Health, Karolinska Institutet, Stockholm, Sweden; Institute of Environmental Medicine, Karolinska Institutet, Stockholm, Sweden; Centre for Occupational and Environmental Medicine, Region Stockholm, Stockholm, Sweden; Institute of Environmental Medicine, Karolinska Institutet, Stockholm, Sweden; Department of Public Health Sciences, Stockholm University, Stockholm, Sweden

## Abstract

Social inequalities in alcohol-related morbidity and mortality are well-established, but the reasons are not fully understood. One possible reason is labor market difficulties stemming from lower educational qualifications, leading to alcohol-related harm. The present study aims to investigate the extent to which differences in labour market marginalization (LMM) (including differences in timing and type of LMM) explain educational differences in alcohol-related mortality, and whether this is independent of pre-labor market selection factors. A register-based cohort study included all men born between 1949 and 1951 who underwent Swedish military conscription in 1969/70 and were alive at age 55 (*n = *45 168). Nationwide registers provided data on education level and alcohol-related mortality. LMM was measured by unemployment, sickness absence, and disability pension. Pre-labor market factors included health behaviors, cognitive ability, and health from conscription exams. Cox regression analyses were used to obtain hazard ratios (HR) with 95% confidence intervals (CI). The explanatory role of LMM was assessed by percentage attenuation of HR. Men with primary and secondary education had higher risks of alcohol-related mortality (HR: 4.23, HR: 2.92) compared to those with university education. LMM explained a substantial part of these differences (42% and 37%). However, LMM's effect was smaller (18% and 7%) when pre-labor market factors were considered. Men with lower education levels in Sweden are more likely to die from alcohol-related causes compared to higher educated men. While differences in LMM contribute to these disparities, its explanatory power diminishes when considering pre-labor market factors, suggesting potential selection effects.

## Introduction

It is well-established in the literature that social inequalities in alcohol-related morbidity and mortality exist, such that individuals with lower socioeconomic position (SEP) are at an increased risk of alcohol-related health problems [1]. Groups with low SEP have a four-fold increased risk of alcohol-attributable mortality compared to groups with higher SEP, which is substantially higher compared to the two-fold increased risk found concerning all-cause mortality [1]. The underlying reasons for these differences are not fully understood as there could be several different multifaceted mechanisms at play [2, 3]. One possible reason could be difficulties in the labor market which can result from lower educational qualifications and lead to alcohol-related harm. Given that lower levels of education could lead to worse labor market position which in turn increases the risk of alcohol-related harm, one such factor that could be of importance is labour market marginalization (LMM) or disrupted work histories.

LMM refers to being distant from the labour force because of sickness absence, unemployment, or disability pension. Evidence suggests that individuals with lower SEP experience more frequent and longer spells of sickness absence and unemployment across working life compared to individuals with high SEP [4, 5]. Furthermore, individuals with lower SEP are at an increased risk of disability pension [6]. Consequently, individuals with lower SEP have a weaker attachment to the labor force and are more often in lower-paid employment. This could be considered a stressful experience, potentially triggering unhealthy coping strategies such as increased alcohol consumption [7]. In addition, in cases of long-term unemployment, sickness absence, or disability pension, the social control mechanisms present in the workplace, the daily structure, and the social support that typically prevent harmful drinking behavior become compromised [8]. Furthermore, previous research has found that unemployment is associated with poor health and premature all-cause and cause-specific mortality [9, 10]. According to a recent meta-analysis, being unemployed is associated with more than a three-fold increased risk of alcohol-related mortality compared to being employed [10]. An increased risk of all-cause and cause-specific mortality, including alcohol-related mortality, has also been found among individuals who have experienced bouts of sickness absence and disability pension [11, 12]. Lastly, the timing of LMM could be of importance. For example, young adults who have recently entered the labor market are more vulnerable than those who have been established for longer, as they lack both work experience and social security benefits in case of unemployment and sickness absence. In addition, research suggests that older adults may be more vulnerable in the labor market, facing greater challenges in returning after job loss or sickness absence [13, 14]. Research looking at LMM as an explanation for the socioeconomic differences in alcohol-related mortality is scarce. Given that employment histories are today more de-standardized and more fragmented than before [15, 16] this could be of importance.

Furthermore, given the potential selection effects, it is important to account for pre-labor market factors. Previous research shows that early life factors and differential drinking patterns partly explain the social inequalities in alcohol-related mortality. For example, childhood SEP and cognitive ability are factors that predict later SEP and health, indicating that there are selection effects that could partly explain the social gradient in health [17–19]. In addition, previous research has found a positive association between cognitive ability measured before labour market entry and later alcohol-related morbidity and mortality [20]. Furthermore, evidence suggests that differences in levels and patterns of drinking (i.e. heavy episodic drinking) between high and low SEP groups explain about 25% of the social gradient in alcohol-related harm [2, 21–25]. These factors explain a large part of the social gradient in alcohol-related mortality, but not all [24, 26]. Consequently, research is needed looking at other factors that are predictive of alcohol-related mortality and that vary between SEP groups.

Thus, the present study aims to investigate the extent to which differences in LMM (including differences in timing and type of LMM) explain educational differences in alcohol-related mortality, and whether this is independent of pre-labor market selection factors. The highest level of education is used to estimate SEP because it is usually established before labor market entry and remains stable over time. Other measures of SEP such as income and occupation may be subject to reverse causation as they may be affected by alcohol use over time [27]. To this end, we will follow a large cohort of men in high-quality nationwide registers. Using a cohort with information from late adolescence makes it possible to take early established risk factors into account to control for potential health-related selection into educational groups.

## Methods

### Study population

The current study was based on a cohort of men born between 1949 and 1951 in Sweden who underwent Swedish military conscription examination in 1969/70 (*n = *49 132). At that time, military service was obligatory by law for males aged 18–20 years in Sweden. Individuals with severe handicaps or congenital disorders were exempted from conscription (2%–3% of the general population). The conscription cohort has been linked to various high-quality nationwide registers [28]. Thus, the cohort includes information from conscription/young adulthood, LMM throughout working life up to the age of 55 years and alcohol-related mortality up to the age of 70 years. The age of 55 was selected as the start of follow-up as we were interested in the effects of LMM across working life and severe alcohol-related health problems are more common in later adulthood [29].

**Figure 1.**
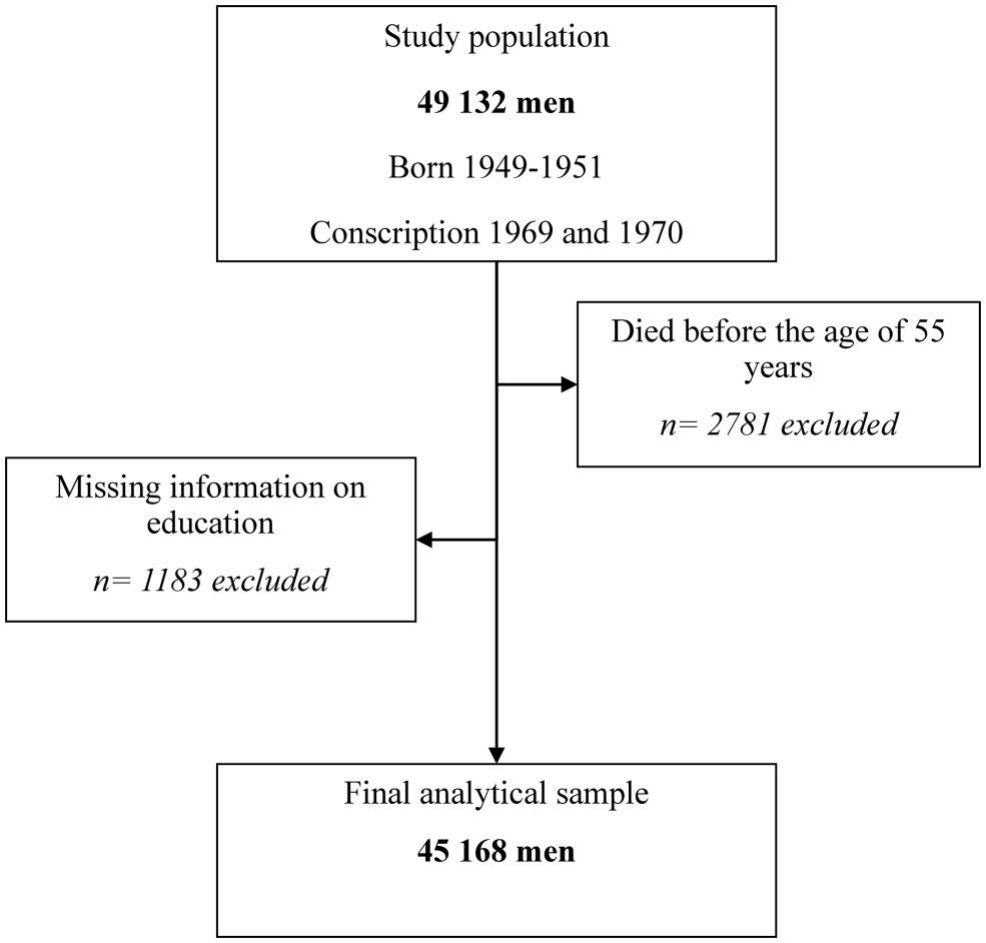
Flow chart describing the selection process of the analytical sample.

Men who died before the age of 55 (*n = *2781) or had missing information on educational attainment at age 55 (*n = *1183) were excluded from the study population (Fig. 1). Generally, excluded men had a lower cognitive ability, worse lifestyle habits, and health during late adolescence, and were to a greater extent marginalized from the labor force during working life compared to the men included in the study (Supplementary Table S1). The final analytical sample consisted of 45 168 men. The study was approved by the Swedish Research Ethics Authority (Dnr: 2018-01917).

### Measures

This study investigates the association between education (exposure) and alcohol-related mortality (outcome) while measuring the extent to which this relationship is explained by labour marker marginalization (explanatory factors), while also accounting for pre-labor market confounding factors (confounders).

### Outcome: alcohol-related mortality

Information on alcohol-related mortality was obtained from the Cause of Death Register from the age of 55 years (2004–6) until the end of 2019. The register includes diagnosis information according to the Swedish version of the International Statistical Classification of Diseases version 10 (from 1997). Several ICD codes were used to identify alcohol-related diagnoses, as either the underlying or contributing cause of death. These include alcohol dependency, alcoholic liver disease, and toxic effect of alcohol (E24.4, F10, G31.2, G62.1, G72.1, I42.6, K29.2, K70, K85.2, K86.0, O354, T51, X45, X65, Z502, Z714, Z721, Y90, Y91).

### Exposure: education

Information on the highest level of educational qualification was obtained from the Longitudinal Register of Education and Labour Market Statistics (LISA) at baseline, the year the men turned 55 years old (2004/2005/2006). It is assumed that this represents earlier educational attainment as the majority complete their highest level of education by age 25 [30]. The highest level of education was categorized into three separate groups based on the number of years in education: primary (<9 years), upper secondary (10–12 years), and university (≥13 years), the latter group being the reference category.

### Explanatory factors: labor market marginalization

To explain the educational differences in alcohol-related mortality several explanatory factors related to labor market marginalization were chosen. In line with previous research, three dimensions of labor market marginalization were included in the current study; unemployment, sickness absence, and disability pension [31]. In line with our previous research, four measures of unemployment were included—youth unemployment, unemployment in young adulthood, unemployment in middle adulthood, and unemployment in older adulthood [5, 32]. Information on youth unemployment (before the age of 18 years) was collected from the conscription examination where the enlistees were asked to identify if they had been unemployed at least 3 months after finishing school. Unemployment in young adulthood was obtained between 1974 and 1991 when the men were 23/24/25 years old and 40/41/42 years old. Information on unemployment in young adulthood was collected from the Swedish Income and Tax Register upon receiving unemployment benefits (Unemployment Insurance Funds) or unemployment assistance (Social Insurance Agency) and was defined as receiving any unemployment benefit or assistance for at least 4 out of the 15 years, as has been done previously [5, 32]. The LISA register was used to identify individuals who had registered at least 180 days of unemployment annually in middle adulthood (between 1992 and 1998/1999/2000, when the men were aged 41/42/43 years to 49 years) and older adulthood (between 1999/2000/2001 and 2003/2004/2005, when the men were aged 50 to 54 years).

Also, in line with previous research, long-term sickness absence was defined as receiving sickness benefits from the Swedish Social Insurance Agency for at least 90 days during one calendar year [31, 32]. The LISA register was used to identify individuals who had any registered long-term sickness absence spells in middle adulthood (between 1994 and 1998/1999/2000, when the men were 43/44/45 years to 49 years) and older adulthood (between 1999/2000/2001 and 2003/2004/2005, when the men were aged 50–54 years).

Information on disability pensions was collected from the LISA register and defined in accordance with previous research [31, 32]. Men who were granted either full-or part-time disability pension before the age of 49 years (1998/1999/2000 and earlier) were defined as being disability pension beneficiaries in middle adulthood and men who received disability for the first time between 1999/2000/2001 and 2003/2004/2005, when the men were aged 50–54 years were considered as being on disability pension in older adulthood. Due to register constraints, the age cut-offs vary across the three different measures of LMM.

### Confounders

Several covariates were chosen to represent pre-labor market factors potentially related to selection into education and alcohol-related outcomes. Childhood SEP was defined using their father’s occupational class and obtained when the men were 9–11 years old from the 1960 Swedish National Population and Housing Censuses. Using the Swedish socioeconomic classification of occupations childhood SEP was categorized into seven groups (i) unskilled workers, (ii) skilled workers, (iii) low-level non-manual employees, (iv) intermediate non-manual employees, (v) high-level non-manual employees, (vi) self-employed or farmers, and (vii) those not classified into a socioeconomic group [33].

*Screening results from conscription*: from the conscription examination we also derived information on the men’s cognitive ability, health behaviors, and health which were included in the analyses as potential confounders. Cognitive ability was measured using the scores of an IQ test administered to the enlistees. The test included four subtests designed to capture their logical, spatial, verbal and technical abilities [34]. From these four subtests, a global IQ score based on Stanine scores was calculated and categorized into high, medium, and low score for the analyses. In addition, information on smoking (≥5 cigarettes/day), risky use of alcohol (consuming 250 g of 100% alcohol per week or more, having been detained because of drunkenness, using alcohol as an eye-opener, or being drunk often), BMI ≥25 and low emotional control (low-stress tolerance and/or anxiety, reduced functioning due to psychosomatic symptoms and uncontrollable nervousness, anxiousness, or aggression) was also derived from the conscription examination. Information on mental and physical health was also obtained from the conscription examination. Psychiatric and musculoskeletal diagnoses were diagnosed by a physician and psychiatrist and defined according to the ICD-8; codes 290–315 and 710–738, respectively.

### Statistical analysis

Pearson’s χ^2^ test was used to test the descriptive differences of the study population. The association between educational qualification and alcohol-related mortality was estimated using Cox proportional hazard models to obtain hazard ratios (HR) with 95% confidence intervals (CI). The proportional hazards assumption was tested using Schoenfeld residuals and found not to be violated (all *P* values ≥ .05). Person time was calculated from age 55 years (1 January 2004, at the earliest) until the date of death, date of emigration, or until the end of follow-up (31 December 2019), whichever came first. Follow-up began at age 55 years to reduce health-related selection bias among older adults in the labor market, while allowing for an extended period of potential exposure to labor market marginalization.

In the analysis of educational differences in alcohol-related mortality, a crude model was first fitted. In the first model adjustments for early factors (childhood SEP and screening results from conscription) were made. In subsequent models, all measures of LMM were included individually as well as grouped together. In the final model, all potential explanatory factors and covariates were included concurrently.

Percent attenuation in risk estimates for the different levels of education after the inclusion of the risk factor in question was calculated to determine the contribution of explanatory factors in explaining the education–mortality association. The percentage of HR reduction compared to the crude model was calculated using the formula ((HR_crude_ − HR_adjusted_)/(HR_crude_ − 1)) × 100.

To assess the importance of each early factor, additional analyses were conducted with stepwise inclusion of all covariates from childhood and screening results from conscription. Furthermore, additional analyses were performed excluding cognitive ability as a potential explanatory variable, since education and cognitive ability are highly correlated [35]. Missing values for the baseline variables were coded as separate categories, as the same conclusions were reached when excluding observations with missing data (Supplementary Table S2). All analyses were computed using Stata Statistical Software: Release 17.

## Results

### Baseline characteristics

The baseline characteristics of the study population, stratified by the highest attained level of education, are shown in Table 1. Men with lower levels of education had generally a lower level of childhood SEP, lower cognitive ability and emotional control, worse health and health behaviors, and a higher prevalence of unemployment, sickness absence, and disability pension across their working lives compared to men with university-level education.

**Table 1.** Baseline characteristics of study population, stratified by level of education

	University, *n* (%)	Upper secondary, *n* (%)	Primary, *n* (%)	*P* value
Total	13 990 (31.0)	20 462 (45.3)	10 716 (23.7)	
Childhood SEP^a^				<.001
Unskilled worker	3020 (21.6)	7300 (35.7)	4617 (43.1)	
Skilled worker	2382 (17.0)	4827 (24.1)	2426 (22.6)	
Low-level non-manual employee	1966 (14.1)	1986 (9.7)	659 (6.2)	
Intermediate non-manual employee	3743 (26.8)	2956 (14.6)	828 (7.7)	
High-level non-manual employee	1453 (10.4)	674 (3.3)	189 (1.8)	
Farmer	1181 (8.5)	2153 (10.5)	1725 (16.1)	
Not classified	245 (1.8)	466 (2.3)	272 (2.5)	
Cognitive ability^b^				<.001
High (7–9)	8615 (61.6)	4843 (23.7)	930 (8.7)	
Medium (4–6)	4979 (35.6)	11 731 (57.3)	5566 (51.9)	
Low (1–3)	389 (2.8)	3868 (18.9)	4207 (39.3)	
Missing	7 (0.1)	20 (0.1)	13 (0.1)	
Health behaviors^b^				
Smoking ≥5 cigarettes/day	4443 (31.8)	10 264 (50.2)	6042 (56.4)	<.001
Risky use of alcohol	2038 (15.5)	4605 (22.4)	2747 (25.6)	<.001
BMI ≥25	608 (4.4)	1360 (6.7)	926 (8.6)	<.001
Low emotional control^b^	3392 (24.3)	6080 (29.7)	3919 (36.6)	<.001
Psychiatric diagnosis^b^	1091 (7.8)	2380 (11.6)	1716 (16.0)	<.001
Musculoskeletal diagnosis^b^	2330 (16.7)	3370 (16.5)	1911 (17.8)	<.001
Unemployment				
Youth UE^b^	507 (3.6)	2648 (12.9)	2263 (21.1)	<.001
UE in young adulthood^c^	521 (3.7)	1610 (7.9)	613 (5.7)	<.001
UE in middle adulthood ^d^	1250 (8.9)	4042 (19.8)	1770 (16.5)	<.001
UE in older adulthood^e^	725 (5.2)	1734 (8.5)	696 (6.5)	<.001
Sickness absence				
Long-term SA in middle adulthood^f^	382 (2.7)	1434 (7.0)	851 (7.9)	<.001
Long-term SA in older adulthood^e^	1170 (8.4)	2989 (14.6)	1747 (16.3)	<.001
Disability pension				
DP in middle adulthood^g^	251 (1.8)	1163 (5.7)	887 (8.3)	<.001
DP in older adulthood^e^	363 (2.6)	1091 (5.3)	653 (6.1)	<.001
Outcome^h^				
Alcohol-related mortality	69 (0.5)	275 (1.3)	202 (1.9)	<.001

a Measured in 1960.

b Measured during conscription in 1969.

c Measured from 1974 to 1991.

d Measured from 1992 to1998/1999/2000.

e Measured from 1999/2000/2001–2003/2004/2005.

f Measured from 1994 to1998/1999/2000.

g Measured 1998/1999/2000 and earlier.

h Measured from the age of 55 years.

SEP = socioeconomic position, BMI = body mass index, UE = unemployment, SA = sickness absence, DP = disability pension.

During follow-up, a total of 546 (1.2%) men died due to alcohol-related diagnoses. Follow-up time was on average 13.8 years. All potential risk factors included in the model, except for BMI, were positively associated with an increased risk of alcohol-related mortality, as seen in Table 2.

**Table 2.** Unadjusted hazard ratios (HRs) and 95% confidence intervals (CIs) on the association between each potential risk factor and alcohol-related mortality

	Alcohol-related mortality
	HR (95% CI)^a^
Childhood SEP	
Unskilled worker	3.69 (1.96–6.95)
Skilled worker	2.92 (1.53–5.57)
Low-level non-manual employee	2.70 (1.37–5.31)
Intermediate non-manual employee	2.63 (1.37–5.07)
High-level non-manual employee (ref)	1.00
Farmer	1.55 (0.76–3.15)
Cognitive ability	
High (7–9) (ref)	1.00
Medium (4–6)	1.98 (1.57–2.49)
Low (1–3)	3.01 (2.34–3.88)
Health behaviors	
Smoking	2.65 (2.21–3.18)
Risky use of alcohol	2.52 (2.12–3.00)
BMI ≥25	1.00 (0.70–1.41)
Low emotional control	1.67 (1.41–1.98)
Psychiatric diagnosis	1.95 (1.58–2.41)
Musculoskeletal diagnosis	1.19 (0.96–1.47)
Unemployment	
Youth UE	1.77 (1.43–2.20)
UE in young adulthood	2.32 (1.81–3.00)
UE in middle adulthood	3.41 (2.86–4.05)
UE in older adulthood	2.90 (2.32–3.62)
Sickness absence	
Long-term SA in middle adulthood	3.27 (2.60–4.11)
Long-term SA in older adulthood	2.91 (2.42–3.50)
Disability pension	
Disability pension in middle adulthood	4.54 (3.65–5.65)
Disability pension in older adulthood	3.19 (2.48–4.11)

a HR = hazard ratio, SEP = socioeconomic position, BMI = body mass index, UE = unemployment, SA = sickness absence.

### Alcohol-related mortality

A graded association between the level of education and alcohol-related mortality was found. Men with primary level education had more than a four-fold increased risk and men with upper secondary level education had almost a three-fold increased risk of alcohol-related mortality compared to men with the highest level of education (Table 3).

**Table 3.** Crude and adjusted hazard ratios (HRs) with 95% confidence intervals (CIs) for the association between highest level of education and alcohol-related mortality (546 events)

	University	Upper secondary		Primary	
	HR (95% CI)	HR (95% CI)^a^	% Attenuation	HR (95% CI)	% Attenuation
Crude	1.00	2.92 (2.24–3.80)		4.23 (3.23–5.57)	
Adjusted for all early factors	1.00	2.11 (1.59–2.80)	42	2.69 (1.98–3.66)	48
Youth unemployment	1.00	2.82 (2.16–3.68)	5	3.97 (3.01–5.25)	8
Unemployed in young adulthood	1.00	2.80 (2.15–3.65)	6	4.14 (3.15–5.45)	3
Unemployed in middle adulthood	1.00	2.48 (1.90–3.24)	23	3.76 (2.85–4.95)	15
Unemployed in older adulthood	1.00	2.78 (2.13–3.61)	7	4.15 (3.15–5.46)	3
Adjusted for all unemployment	1.00	2.39 (1.83–3.113)	27	3.68 (2.78–4.86)	17
Sickness absence in middle adulthood	1.00	2.73 (2.09–3.56)	10	3.89 (2.95–5.13)	10
Sickness absence in older adulthood	1.00	2.69 (2.06–3.51)	12	3.81 (2.89–5.02)	13
Adjusted for all sickness absence	1.00	2.61 (2.00–3.40)	16	3.66 (2.78–4.83)	18
Disability pension in middle adulthood	1.00	2.66 (2.04–3.47)	13	3.66 (2.77–4.83)	18
Disability pension in older adulthood	1.00	2.80 (2.15–3.65)	6	4.00 (3.05–5.27)	7
Adjusted for all disability pension	1.00	2.52 (1.93–3.29)	21	3.40 (2.58–4.50)	26
Adjusted for all measures of labour market marginalization	1.00	2.11 (1.61–2.76)	42	3.02 (2.28–4.01)	37
Full model	1.00	1.77 (1.33–2.35)	60	2.46 (1.81–3.35)	55

a HR = hazard ratio; % attenuation = representing the proportion of the education–mortality association explained by the risk factor in question.

Previous unemployment had varying attenuation effects on the risk estimates, where unemployment during middle adulthood appears to explain the largest part of the association between education and alcohol-related mortality among males with primary-level education (15%) (Table 3). The explanatory role of sickness absence on the association between education and alcohol-related mortality was similar for both sickness absence in middle adulthood and older adulthood for men with a primary level of education (10%−13%). Whereas disability pension in middle adulthood appears to explain a larger proportion of increased risk of alcohol-related mortality among men with a primary level of education (18%). All measures of LMM together explained a substantial part of the association between education and alcohol-related mortality among individuals with a primary and upper-secondary level of education (37% and 42%, respectively). The graded association between education and alcohol-related mortality remained in the final model, where men with primary level education had a 2.5-fold (95% CI 1.81 − 3.35) and men with upper secondary level education had a 1.8-fold (95% CI 1.33 − 2.35) increased risk compared to men with university level education.

Risk factors measured before labour market entry explained a large part (48% and 42%, respectively) of the increased risk, compared to the crude model (Table 3).

In the full model, the early factors and all labor market marginalization factors together attenuated the association by 55%−60%. After adding the early factors into the model, the explanatory role of LMM was substantially smaller, 7% (55% − 48%) for primary level education and 18% (60% − 42%) for upper secondary education.

### Additional analysis

In the additional analyses, when all the covariates were entered stepwise, adjusting for cognitive ability appeared to have the greatest effect on the risk estimates, and risky use of alcohol during adolescence attenuated the risk estimate by 13% among men with primary-level education (Supplementary Table S3). Excluding cognitive ability as an explanatory variable in the main analyses led to less attenuation in the adjusted model (48% of the difference was explained when cognitive ability was included versus 33% when it was not included among men with primary education) (Supplementary Table S4).

## Discussion

The results of this study suggest that LMM, especially during middle adulthood, explained a substantial part of the risk difference in alcohol-related mortality. However, after taking into consideration factors before labour market entry, LMM had less effect on the risk estimates found among men with lower levels of education.

In line with previous research, we found educational differences in alcohol-related mortality where men with primary level education had an almost four-fold increased risk compared to men with the highest level of education [1]. The role of labor market marginalization in explaining the educational differences in alcohol-related mortality appeared to be of less importance among men with lower levels of education compared to men with upper secondary level education. In line with previous research [4, 5], we found that men with a primary level of education were to a greater extent marginalized from the labor market compared to men with higher levels of education. Furthermore, in line with previous research, we found that each measure of LMM was an independent risk factor for alcohol-related mortality [10]. The different measures of LMM and their timing in the life course were found to have differing effects on the risk estimates among men with lower levels of education. Being marginalized from the labour market during middle adulthood appeared to explain a larger part of the educational differences in alcohol-related mortality compared to earlier and later marginalization. A potential reason for this could be that individuals after the age of 45 years appear to be more vulnerable in the labour market and face greater difficulty returning after job loss and sickness absence [13, 14]. Furthermore, disability pension explained slightly more of the educational differences compared to the other two measures of LMM which could be due to disability pension being more strongly related to poor health and mortality [36]. Much of the explanatory power of LMM diminished when taking the early factors into account. A likely reason for this could be that much of the risk differences can be explained by selection effects [17], which has not been taken into consideration in previous work looking at the association between employment status and alcohol-related mortality [10].

Factors measured before labor market entry, i.e. during childhood and late adolescence, appeared to explain the largest part of the educational differences in alcohol-related mortality. These findings strengthen the notion of the importance of taking into account early factors when studying social inequalities, to ensure one is not overestimating the effects of the disrupted employment histories [17]. For example, we found that cognitive ability was particularly important in explaining this relationship. Previous research has found that low cognitive ability is a strong predictor for unsuccessful educational achievement at age 30 [37] and alcohol-related morbidity and mortality [38], suggesting that there could be some selection effect that might explain part of the social gradient in health [17–19]. Furthermore, we found that risky use of alcohol measured during conscription was more prevalent among men with lower levels of education which is in line with previous research [21–25]. When exploring the role of risky alcohol use on the educational differences between alcohol-related mortality, we found that around 13% of the association was explained, which is smaller compared to previous research [2]. A potential explanation for this could be that we included a measure of risky use of alcohol during late adolescence even though this is likely to change later in the life course. Given that labor market marginalization could be a risk factor for increased alcohol consumption, the difference in risky alcohol consumption between levels of education could be greater in adulthood [7].

### Strengths and limitations

A major strength of this study was the opportunity to follow a large cohort of men across their working life in nationwide registers, as this decreases the risk of self-report bias and attrition [39]. Another major strength was the inclusion of factors from childhood and adolescence that are strong determinants of educational qualifications. Not including these increases the risk of overestimating the role of explanatory factors later in life when explaining social inequalities in mortality [17]. In other words, this is a way of dealing with health selection operating in educational attainment. Furthermore, we included several measures of labor market marginalization across working life as opposed to one point in time only. This is a strength as experiencing unemployment, sickness absence or disability pension appears to have different effects depending on when it happens.

Several important limitations of our study need to be acknowledged when interpreting the results. First, an obvious limitation is that our study only includes men. The conscription cohort was utilized in this study due to its completeness on early life risk factors of interest as previous research suggests that early risk factors are of importance when explaining social inequality in mortality [17]. If we had had the opportunity to include women as well, the results likely would have been slightly different as previous literature suggests that the association between SEP and alcohol-related mortality is slightly stronger for men compared to women [40]. Furthermore, we were only able to include one measure of alcohol consumption which was registered during late adolescence and consequently, we do not have information on the men’s alcohol consumption later in life. This is a limitation, as previous research trying to explain the socioeconomic inequalities in alcohol-related mortality has found that frequency and especially pattern of alcohol consumption explains a substantial part of the inequalities [2]. As alcohol use can lead to difficultly sustaining and maintaining employment [7] we have included a measure of pre-labour alcohol use; however, it is difficult to disentangle to what extent later alcohol use contributes to an increased risk of LMM. Also, other types of LMM (i.e. underemployment, informal work, and job precarity) that are not included in the social insurance system, but could be of interest, were not captured in this study. Lastly, these findings may not apply to countries with different social security systems.

## Conclusion

The results of this study show that men in Sweden with primary and upper secondary level education are more likely to die due to an alcohol-related diagnosis compared to same-aged higher educated men. While differences in labor market marginalization, especially in middle adulthood, contribute to these educational disparities, much of their explanatory power diminished when considering factors measured before labor market entry, suggesting potential selection effects.

## Supplementary Material

ckaf163_Supplementary_Data

## Data Availability

The data that support the findings of this study are available from Statistics Sweden but restrictions apply to the availability of these data, which were used under license for the current study, and so are not publicly available. Data are however available from the authors upon reasonable request and with permission of Statistics Sweden.
