# Supplementary material for: Level of education, labor-market marginalization, and alcohol-related mortality: a cohort study of Swedish men
Source: Eur J Public Health. 2025 Sep 25;35(6):1241–7. doi: 10.1093/eurpub/ckaf163 (PMC12707506; doi:10.1093/eurpub/ckaf163)
Supplement: ckaf163_Supplementary_Data [file ckaf163_supplementary_data.docx]

Supplementary Table 1: Baseline characteristics of the individuals included and excluded in the study population

|  | Included  n(%) | Excluded  n(%) | p-value |
| --- | --- | --- | --- |
| Total | 45 168 (91.9) | 3964 (8.1) |  |
| Childhood SEP^a^  Unskilled worker  Skilled worker  Low-level non-manual employee  Intermediate non-manual employee  High-level non-manual employee  Farmer  Not classified | 14 937 (33.1)  9735 (21.6)  4611 (10.3)  7527 (16.7)  2316 (5.1)  5059 (11.2)  983 (2.2) | 1338 (33.8)  779 (19.7)  376 (9.5)  749 (18.9)  255 (6.4)  343 (8.7)  124 (3.1) | <0.001 |
| Cognitive ability^b^  High (7-9)  Medium (4-6)  Low (1-3)  Missing | 14 388 (31.9)  22 276 (49.3)  8464 (18.7)  40 (0.1) | 1169 (29.5)  1883 (47.5)  911 (23.0)  1 (0.0) | <0.001 |
| Health behaviors^b^  Smoking ≥5 cigarettes/day  Risky use of alcohol  BMI ≥25 | 20 749 (45.9)  9390 (20.8)  2894 (6.4) | 2201 (55.5)  1094 (27.6)  334 (8.4) | <0.001  <0.001  <0.001 |
| Low emotional control^b^ | 13 391 (29.7) | 1479 (37.3) | <0.001 |
| Psychiatric diagnosis^b^ | 5187 (11.5) | 730 (18.4) | <0.001 |
| Musculoskeletal diagnosis^b^ | 7611 (16.9) | 688 (17.4) | <0.001 |
| Inpatient-care psychiatric diagnosis^c^ | 2895 (6.4) | 1031 (26.0) | <0.001 |
| Employment histories  Youth unemployment^b^  Unemployed in young adulthood^d^  Unemployed in middle adulthood^e^  Unemployed in older adulthood^f^ | 5418 (12.0)  2744 (6.1)  7062 (15.6)  3155 (7.0) | 718 (18.1)  299 (7.5)  507 (12.8)  69 (1.7) | <0.001  <0.001  <0.001  <0.001 |
| Sickness absence  Long-term sickness absence in middle adulthood^g^  Long-term sickness absence in older adulthood^f^ | 2667 (5.9)  5906 (13.1) | 337 (8.5)  320 (8.1) | <0.001 |

SEP: socioeconomic position, BMI: Body mass index

^a^Measured in 1960

^b^Measured during conscription in 1969

^c^Measured from 1971 to 2003/2004/2005

^d^Measured from 1974 to 1991

^e^Measured from 1992 to1998/1999/2000

^f^Measured from 1999/2000/2001 to 2003/2004/2005

^g^ Measured from 1994 to1998/1999/2000

Supplementary Table 2. Complete case analyses excluding 2043 individuals with missing information on covariates. Crude and adjusted hazard ratios (HRs) with 95% confidence intervals (CIs) for the association between highest level of education and alcohol-related mortality.

|  | University | Upper secondary |  | Primary |  |
| --- | --- | --- | --- | --- | --- |
|  | HR (95%CI) | HR (95%CI) | % Attenuation | HR (95%CI) | % Attenuation |
| Crude | 1.00 | 2.87 (2.19, 3.75) |  | 4.22 (3.19, 5.58) |  |
| **Adjusted for all early factors** | 1.00 | 2.09 (1.57, 2.79) | 41 | 2.71 (1.98, 3.71) | 47 |
| Youth unemployment | 1.00 | 2.78 (2.12, 3.64) | 5 | 3.97 (2.99, 5.27) | 8 |
| Unemployed in young adulthood | 1.00 | 2.75 (2.10, 3.60) | 7 | 4.13 (3.12, 5.46) | 3 |
| Unemployed in middle adulthood | 1.00 | 2.43 (1.86, 3.19) | 23 | 3.75 (2.83, 4.97) | 14 |
| Unemployed in older adulthood | 1.00 | 2.73 (2.09, 3.58) | 7 | 4.14 (3.13, 5.48) | 2 |
| **Adjusted for all unemployment** | 1.00 | 2.36 (1.79, 3.09) | 27 | 3.68 (2.77, 4.89) | 17 |
| Sickness absence in middle adulthood | 1.00 | 2.69 (2.05, 3.52) | 10 | 3.89 (2.94, 5.15) | 10 |
| Sickness absence in older adulthood | 1.00 | 2.65 (2.03, 3.48) | 11 | 3.80 (2.87, 5.04) | 13 |
| **Adjusted for all sickness absence** | 1.00 | 2.57 (1.96, 3.37) | 16 | 3.66 (2.76, 4.85) | 17 |
| Disability pension in middle adulthood | 1.00 | 2.62 (2.00, 3.43) | 13 | 3.64 (2.74, 4.83) | 18 |
| Disability pension in older adulthood | 1.00 | 2.76 (2.11, 3.61) | 6 | 4.01 (3.03, 5.30) | 7 |
| **Adjusted for all disability pension** | 1.00 | 2.48 (1.89, 3.26) | 21 | 3.40 (2.45, 4.51) | 25 |
| **Adjusted for all measures of labour market marginalization** | 1.00 | 2.08 (1.58, 2.73) | 42 | 3.03 (2.28, 4.03) | 37 |
| **Full model** | 1.00 | 1.76 (1.32, 2.34) | 60 | 2.50 (1.83, 3.42) | 53 |

HR, Hazard ratio; % attenuation, representing the proportion of the education–mortality association explained by the risk factor in question. Early factors: childhood SEP and screening results from conscription.

Supplementary Tabel 3. Crude and adjusted hazard ratios (HRs) with 95% confidence intervals (CIs) for the association between highest level of education and alcohol-related mortality (546 events) (stepwise adjustment for early factors)

|  | University | Upper secondary |  | Primary |  |
| --- | --- | --- | --- | --- | --- |
|  | HR (95%CI) | HR (95%CI) | % Attenuation | HR (95%CI) | % Attenuation |
| Crude | 1.00 | 2.92 (2.24, 3.80) |  | 4.23 (3.23, 5.57) |  |
| Childhood SEP (1960) | 1.00 | 2.84 (2.17, 3.72) | 4 | 4.22 (3.18, 5.60) | 0 |
| Cognitive ability (1969) | 1.00 | 2.48 (1.88, 3.28) | 23 | 3.27 (2.42, 4.42) | 30 |
| Smoking (1969) | 1.00 | 2.51 (1.92, 3.28) | 21 | 3.47 (2.63, 4.59) | 23 |
| Risky use of alcohol (1969) | 1.00 | 2.70 (2.07, 3.52) | 11 | 3.79 (2.88, 5.00) | 13 |
| BMI (1969) | 1.00 | 2.92 (2.24, 3.81) | 0 | 4.25 (3.23, 5.60) | +1 |
| Low emotional control (1969) | 1.00 | 2.85 (2.18, 3.71) | 4 | 4.00 (3.04, 5.27) | 7 |
| Psychiatric diagnosis (1969) | 1.00 | 2.58 (1.98, 3.36) | 18 | 3.53 (2.68, 4.65) | 22 |
| Musculoskeletal diagnosis (1969) | 1.00 | 2.92 (2.24, 3.80) | 4 | 4.22 (3.21, 5.55) | 7 |

Supplementary Table 4. Crude and adjusted hazard ratios (HRs) with 95% confidence intervals (CIs) for the association between highest level of education and alcohol-related mortality, not taking cognitive ability into account.

|  | University | Upper secondary |  | Primary |  |
| --- | --- | --- | --- | --- | --- |
|  | HR (95%CI) | HR (95%CI) | % Attenuation | HR (95%CI) | % Attenuation |
| Crude | 1.00 | 2.92 (2.24, 3.80) |  | 4.23 (3.23, 5.57) |  |
| Early factors | 1.00 | 2.35 (1.80, 3.09) | 29 | 3.18 (2.38, 4.24) | 33 |
| Full model | 1.00 | 1.85 (1.40, 2.43) | 37 | 2.61 (1.95, 3.50) | 26 |

HR, Hazard ratio; % attenuation, representing the proportion of the education–mortality association explained by the risk factor in question. Early factors: childhood SEP and screening results from conscription.
